# Associating Dietary Sustainability with Health: A Focus on General and Central Adiposity

**DOI:** 10.3390/ijerph23030334

**Published:** 2026-03-07

**Authors:** Mariana Rei, Catarina Campos Silva, Duarte Torres, Colin Sage, Sara S. P. Rodrigues

**Affiliations:** 1 Faculdade de Ciências da Nutrição e Alimentação, Universidade do Porto, Rua do Campo Alegre 823, 4150-180 Porto, Portugal; dupamato@fcna.up.pt (D.T.); c.sage@ucc.ie (C.S.); 2 Unidade de Investigação em Epidemiologia (EPIUnit) do Instituto de Saúde Pública, Universidade do Porto, Rua das Taipas 135, 4050-600 Porto, Portugal; 3 Laboratório para a Investigação Integrativa e Translacional em Saúde Populacional (ITR), Universidade do Porto, Rua das Taipas 135, 4050-600 Porto, Portugal; 4 Faculdade de Medicina, Universidade do Porto, Alameda Prof. Hernâni Monteiro, 4200-319 Porto, Portugal; csilva@med.up.pt

**Keywords:** adults, obesity, sustainability score, sustainable diets, 24 h recalls

## Abstract

This study aims to explore the relationship between the Diet Sustainability Score (DSS) and health outcomes, specifically body mass index (BMI) and waist-to-height ratio (WHtR). Using data from 2287 Portuguese adults in the National Food, Nutrition, and Physical Activity Survey (IAN-AF 2015–2016), DSS was calculated based on four dimensions: health-related nutritional attributes, environmental impact, economic affordability, and sociocultural acceptability. Logistic regression models were used to analyse associations between DSS and general adiposity (BMI classes: under/normal weight vs. overweight/obesity) and central adiposity (WHtR classes: healthy vs. unhealthy central adiposity). Models were adjusted for sex, age, education and physical activity level to control for potential confounders. Higher DSS is associated with reduced odds of overweight/obesity (OR = 0.91, 95%CI: 0.88, 0.94), and unhealthy central adiposity (OR = 0.91, 95%CI: 0.87, 0.95), suggesting that more sustainable dietary patterns are associated with more favourable adiposity profiles. This study highlights the importance of promoting sustainable diets as part of public health strategies aimed at addressing obesity and integrating health, environmental, economic, and sociocultural dimensions for more comprehensive, long-term population health improvements.

## 1. Introduction

Obesity, particularly central adiposity, poses significant challenges to public health due to its strong association with various non-communicable diseases, including cardiovascular disorders, type 2 diabetes, and certain cancers [1,2,3]. Worldwide, the number of adults affected by obesity has more than doubled since 1990, reaching 878 million in 2022 [4]. In the European region, approximately 60% of the adult population is living with overweight or obesity, contributing to an estimated 1.2 million deaths annually [5]. In Portugal, the situation is similarly alarming, with 36.5% of the adult population affected by overweight, 21.6% by obesity, and 50.5% by central obesity in 2015/2016 [6], underscoring a major public health challenge for the country.

The global rising prevalence of obesity has been linked to several risk factors, with diet playing a central role [4,7]. Unhealthy dietary patterns, characterised by high consumption of energy-dense and nutrient-poor food, are major contributors to weight gain and increased central adiposity [7,8]. Given the multifaceted nature of obesity, understanding the influence of diet on body mass index (BMI) and waist-to-height ratio (WHtR) is critical for developing effective public health strategies. Recent evidence suggests that sustainable diets—defined as nutritionally adequate, environmentally friendly, economically viable, and culturally acceptable [9]—may play a key role in preventing health problems [10,11,12,13,14]. However, the relationship between diet sustainability and obesity, particularly in general and central adiposity, remains underexplored [15].

Diet sustainability can be assessed using different methodological approaches. One approach relies on modelled or hypothetical dietary patterns, defined a priori based on official dietary recommendations, traditional dietary patterns, food substitution models, or expert-defined diets, such as the EAT-Lancet reference diet [16]. Another approach focuses on observed dietary patterns, assessed using empirical individual-level food consumption data that reflect diets as they are actually consumed in the population [16]. Although hypothetical diets do not require primary dietary data, they may fail to capture the full diversity of existing dietary patterns that could be similarly or even more sustainable [16]. Accordingly, several widely used sustainability indices, including the Planetary Health Diet Index and the EAT-Lancet score, are largely grounded in such modelled dietary scenarios or global reference values, which may limit their ability to account for national dietary habits, food availability, and sociocultural contexts. Therefore, assessing the sustainability characteristics of existing diets is particularly relevant when evaluating diet-health relationships within specific populations.

This study investigates the association between the Diet Sustainability Score (DSS)—a multicriteria measure based on observed individual-level dietary data, which evaluates the sustainability of Portuguese dietary patterns across health-related nutritional, environmental, economic, and sociocultural dimensions [17]—and BMI and WHtR. By examining how sustainable diets are associated with adiposity-related health outcomes, this research provides valuable insights for public health initiatives that promote healthier and more sustainable dietary practices.

## 2. Methods

### 2.1. Study Design and Participants

This study used data from the most recent National Food, Nutrition, and Physical Activity Survey (IAN-AF), which collected nationwide and regional data on dietary intake and physical activity among the Portuguese general population [18,19]. Data collection spanned from October 2015 to September 2016. The sample was drawn using probabilistic multistage sampling, with the National Health Registry as the sampling frame. Stratification was based on Portugal’s seven statistical geographical regions, including the mainland and the islands. Primary Health Care Units were randomly selected from each region, followed by the random selection of individuals from each Unit, according to age and sex. The sample’s representativeness was confirmed by comparing it with the 2011 Census data from the National Institute of Statistics.

From the original sample of 6553 participants aged 3 months to 84 years, 5811 individuals completed two non-consecutive 24 h dietary recalls, eight to fifteen days apart. Only adults (≥18 years old) with two completed dietary assessments were included in the present study, resulting in a sample of 3852 participants. Further exclusions were applied to pregnant (*n* = 49) and lactating women (*n* = 47), and participants with missing data on weight (*n* = 7), income (*n* = 372), or household members (*n* = 37). Under, plausible, and over-reporters were identified using the Goldberg method [20] and variation coefficients recommended by Black [21]. After excluding under-reporters (*n* = 1014) and over-reporters (*n* = 39), the final sample comprised 2287 participants.

### 2.2. Data Collection

Data collection was conducted by trained fieldworkers with backgrounds in nutrition and dietetics, using the “You eAT&Move” electronic platform. This platform includes three modules: (1) the “You” module, which collected data on sociodemographic status and anthropometric measures; (2) the “eAT24” module for dietary intake; and (3) the “Move” module, which captured physical activity data. Dietary intake data were collected using two non-consecutive 24 h recalls, utilising the multiple-pass method [22], with food portion estimation supported by food pictures. The data was harmonised with the European Food Safety Authority’s FoodEx2 classification system [23,24].

Sociodemographic data: participants’ sex, age, and education were collected. Age was categorised into three groups: younger adults (18–34 years), middle-aged adults (35–64 years), and older adults (65–84 years). Educational attainment was classified based on the highest level of education completed and divided into three categories: ≤6th year of schooling, 7th–12th year of schooling, or >12th year of schooling.

Physical activity data: collected using the short form of the International Physical Activity Questionnaire (IPAQ) and categorised into three levels: inactive, moderately active, and active [25].

Anthropometry data: weight (kg) and height (m) were measured by trained researchers or self-reported. The correlation between self-reported and measured values was strong (ρ = 0.983 for weight, ρ = 0.964 for height), so self-reported data were used when measured data were missing. BMI was calculated as weight (kg) divided by height squared (m^2^) and categorised according to the World Health Organization guidelines into underweight (BMI < 18.5 kg/m^2^), normal weight (BMI = 18.5–24.9 kg/m^2^), overweight (BMI = 25.0–29.9 kg/m^2^), and obesity (BMI ≥ 30.0 kg/m^2^) [26]. Due to the small number of participants classified as underweight (n = 14), this group was combined with the normal weight group, forming an under/normal weight category (BMI < 25.0 kg/m^2^).

Trained researchers also measured waist circumference (cm). WHtR was assessed as waist circumference (cm) divided by height (cm) and categorised as healthy (<0.5) and unhealthy (≥0.5) central adiposity levels [27,28].

### 2.3. Diet Sustainability Score

The DSS was calculated based on the definition of sustainable diets provided by the Food and Agriculture Organization [9], assessing four dimensions: health-related nutritional, environmental, economic, and sociocultural. Each dimension contributed equally to the overall score, which ranged from 4 to 20 points. Based on quintiles, sustainability indicators for each dimension were assigned scores from 1 to 5, with higher scores indicating better performance.

Health-related Nutritional Dimension: two indicators were used: (1) the Nutrient-Rich Diet Index 7.3 (NRDiet7.3), an adaptation of the original NRDiet9.3 [29,30], which evaluates nutrient density; and (2) the percentage of total energy intake (%TEI) from ultra-processed foods (UPF), classified using the NOVA system [31].

Environmental Dimension: the environmental impact of participants’ diets was measured through two indicators—diet-related greenhouse gas emissions (GHGE) and land use (LU). These were calculated using food-specific estimates from the SHARP-Indicators Database [32] and matched with dietary data from the IAN-AF [33].

Economic Dimension: diet affordability was assessed by estimating the proportion of participants’ income devoted to food, using food price data collected from major Portuguese supermarket chains in July 2023 and adjusted to 2015 based on the Harmonised Index of Consumer Prices.

Sociocultural Dimension: the cultural acceptability of diets was determined by the %TEI from culturally accepted foods, identified through the proportion of consumers and their average intake.

Further information on developing and validating the DSS might be found elsewhere [17].

### 2.4. Statistical Analysis

All statistics were performed using the R Software version 4.4.0. A significance level of α = 0.05 was considered in all analyses. After testing the normality of the variables using the Shapiro-Wilk test, descriptive statistics were used to summarise the characteristics of the study sample. To examine gradients in health-related nutritional, environmental, economic and sociocultural indicators across levels of diet sustainability, continuous outcomes were compared across quintiles of the DSS. Linear trends across DSS quintiles were assessed using linear regression models, with DSS quintiles entered as an ordinal variable and adjusted for sex, age, education and level of physical activity.

Logistic regression models examined the association between the DSS and BMI classes (under/normal weight vs. overweight/obesity) and WHtR classes (healthy vs. unhealthy central adiposity). Crude and adjusted odds ratios (OR) and respective 95% Confidence Intervals (CI 95%) were calculated, and the final models were adjusted for sex, age, education and level of physical activity, as these characteristics can influence both the independent variable (DSS) and the health outcomes (BMI and WHtR). Adjusting for these variables allows the model to estimate the relationship between DSS and health outcomes independently of the effects that sex, age, education or physical activity level may have.

Model diagnostics included the assessment of multicollinearity using generalised variance inflation factors (GVIF) and evaluation of model calibration using the Hosmer–Lemeshow goodness-of-fit test. Discriminative ability of the final models was assessed using the area under the receiver operating characteristic curve (AUC), and classification performance was described using sensitivity, specificity and overall accuracy.

### 2.5. Ethical Procedures

This research involved additional processing of previously collected personal data from the IAN-AF, which had previously obtained ethical approval from the National Commission for Data Protection, the Ethical Committee of the Institute of Public Health of the University of Porto, and the Ethical Commissions of each of the Regional Administrations of Health. All participants were asked to provide their written informed consent for participation according to the Ethical Principles for Medical Research involving human subjects expressed in the Declaration of Helsinki and the national legislation. For this study, a pseudo-anonymised database of IAN-AF was obtained.

## 3. Results

Table 1 shows that more than half of the sample were males (52.4% vs. 47.6% females). In terms of age distribution, 23.4% were younger adults (18–34 years), 56.6% middle-aged adults (35–64 years), and 19.9% older adults (65–84 years). Regarding education, 30.6% completed ≤6th year of schooling, 42.8% completed 7th–12th years, and 26.6% completed ≥12 years of schooling. About the physical activity level, almost half of the sample was inactive (48.9%). Among the participants, 58.8% were classified as having overweight or obesity and 65.0% were classified as having unhealthy central adiposity. The mean DSS was 12.00 (95%CI: 11.88, 12.12).

**Figure 1:**
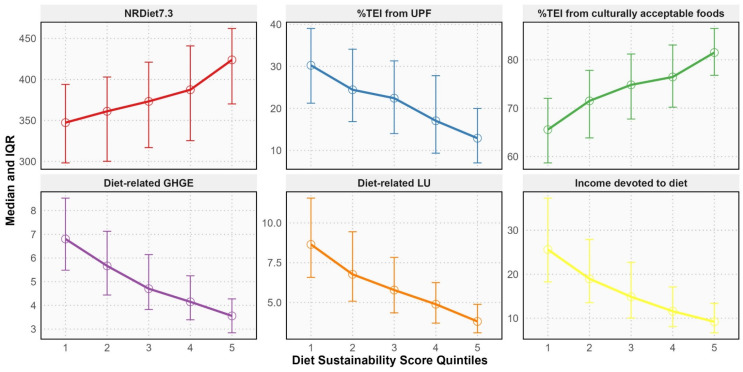
Sustainability indicators across quintiles of the Diet Sustainability Score. IQR, Interquartile Range; NRDiet7.3, Nutrient-Rich Diet Index 7.3; TEI, Total Energy Intake (in kcal); UPF, Ultra-processed foods (according to NOVA classification system); GHGE, Greenhouse-gas emissions (in kgCO_2_eq); LU, Land Use (in m^2^∙year); Income devoted to diet, daily net income allocated to food, in 2015 (in %). Diet Sustainability Score (4–20 points) computed as: *Nutritional score* (1–5 points) + *Environmental score* (1–5 points) + *Economic score* (1–5 points) + *Sociocultural score* (1–5 points), where the *Nutritional score* is the sum of points from *NRDiet7.3* and %*TEI from UPF* indicators × 1/2, the *Environmental score* is the sum of points from *Diet-related GHGE* and *Diet-related LU* indicators × 1/2, the *Economic score* is the sum of points from *Income devoted to diet* indicator × 1, and the *Sociocultural score* is the sum of points from %*TEI from culturally acceptable foods* indicator × 1. All indicators showed significant linear trend across DSS quintiles (*p* for trend < 0.001; adjusted for sex, age, education and physical activity).

Figure 1 shows that higher DSS reflect greater nutrient density, lower intake of UPF, reduced environmental impact in terms of GHGE and LU, greater affordability, and higher cultural acceptability of diets, with statistically significant linear trends across DSS quintiles for all indicators (*p* for trend < 0.001 for all; adjusted for sex, age, education and physical activity).

Table 2 reveals an inverse association between the DSS and BMI and WHtR. Compared to the reference category of BMI (under/normal weight), for each unit increase in the DSS, the odds of being classified as having overweight/obesity significantly decreased by 9%, after adjusting for sex, age, education and level of physical activity. The final model had a fair predictive ability—R^2^ Nagelkerke = 0.215, sensitivity = 81.0%, specificity = 57.0%, AUC = 0.740 (95%CI: 0.719, 0.762), accuracy = 0.710 (95%CI: 0.691, 0.729). Model calibration suggested some lack of fit (Hosmer-Lemeshow *p* < 0.05), indicating that results should be interpreted primarily in terms of association rather than prediction. Although moderate collinearity was observed for DSS and age, all adjusted GVIF^(1/(2·Df)^) values remained below commonly accepted thresholds.

Similarly, for WHtR, the odds of being classified as having unhealthy central adiposity (compared to healthy central adiposity) significantly decreased by 9% for each unit increase in the DSS, after adjusting for sex, age, education and level of physical activity. The final model had a moderate predictive ability—R^2^ Nagelkerke = 0.419, sensitivity = 85.8%, specificity = 63.2%, AUC = 0.841 (95%CI: 0.824, 0.858), accuracy = 0.780 (95%CI: 0.762, 0.797). The model showed good calibration (Hosmer-Lemeshow *p* = 0.95), and no evidence of problematic multicollinearity was observed.

## 4. Discussion

The findings of this population-based study among Portuguese adults indicate that higher diet sustainability, as evaluated by DSS, was associated with lower levels of both general adiposity, as measured by BMI, and central adiposity, as indicated by WHtR. This is consistent with existing literature [11,14]. Notably, the ability of the DSS to predict central adiposity with greater accuracy than general adiposity may reflect the distinct metabolic risks associated with central adiposity. As fat accumulation around the abdominal region is more strongly linked to cardiovascular disease, type 2 diabetes, and other metabolic disorders [34,35,36], it is suggested that promoting sustainable diets could be particularly effective in mitigating these high-risk conditions.

Higher DSS corresponds to nutrient-dense diets, which are high in fruits, vegetables, legumes, and whole grains and tend to have lower energy density and promote satiety, potentially leading to reduced caloric intake and healthier body composition [37,38,39,40]. Additionally, the lower proportion of ultra-processed foods in higher DSS diets likely contributes to reduced central adiposity, as these foods have been consistently linked to excessive calorie intake and increased body weight and fat accumulation, particularly in the abdominal region [41,42,43,44,45]. These dietary characteristics represent plausible mechanistic pathways directly linking higher DSS to lower levels of general and central adiposity. The lower environmental impact of higher DSS diets further highlights how sustainable diets align with better health outcomes and broader ecological goals [46,47,48]. Moreover, the DSS’s cultural acceptability and affordability dimensions suggest that sustainable diets are not only healthier but also accessible and adaptable to the local context, which may promote adherence and long-term health benefits [16,49]. Taken together, the findings of this study indicate that more sustainable dietary patterns are associated with lower prevalence of overweight, obesity, and unhealthy fat distribution in the population, while promoting environmental and social well-being. Given the context of global challenges posed by climate change and rising malnutrition, integrating sustainability into dietary patterns emerges not only as a health and environmental imperative but also an economic and social necessity.

## 5. Strengths and Limitations

This study used the most comprehensive and recent dataset from the IAN-AF, which includes extensive dietary, anthropometric, and sociodemographic information. The large and diverse sample enhances the generalizability of the findings across the Portuguese population. Additionally, the DSS employed provides a thorough assessment of dietary patterns by evaluating nutritional, environmental, economic, and sociocultural dimensions. This multicriteria approach allows for a detailed understanding of how various aspects of diet sustainability are related to general and central adiposity. While these dimensions may not contribute equally to health outcomes, the DSS was designed to capture the overall sustainability profile of dietary patterns, and the examination of dimension-specific effects was beyond the scope of the present study. The study also benefits from rigorous statistical methods, adjusting models for key confounding variables such as sex, age, education, and physical activity level. This helps isolate the effect of diet sustainability on health outcomes. Also, previous studies have primarily focused on the relationship between sustainable diet indexes and BMI or weight gain [15], whereas this study also examines WHtR. While BMI remains a useful tool for estimating health risks, measures like waist circumference may provide a more accurate reflection of body fat distribution and accumulation [50,51].

However, the study has some limitations. Its cross-sectional design restricts the ability to infer causality between diet sustainability and adiposity. Longitudinal studies are necessary to establish causal relationships and to explore the potential mediating factors between sustainable diets and weight regulation over time. Additionally, the reliance on self-reported data for some variables, including anthropometric measures, introduces potential measurement errors or biases, although the correlations between self-reported and measured values were strong. Exclusions due to missing data on key variables, such as income and household members, could affect the external validity of the findings. Sensitivity analyses suggest that missing data had minimal impact, but this remains a consideration. Lastly, although WHtR is strongly correlated with BMI and may therefore provide limited information independent of overall adiposity, it remains a widely used and recommended indicator of central adiposity in population-based research and public health context; alternative composite measures like the Anthropometric Risk Index (ARI) [52], may provide additional insights for estimating health risk (e.g., mortality or cardiovascular disease) based on multiple body measurements. Despite these limitations, the study provides valuable insights into the relationship between diet sustainability and adiposity-related health outcomes. Future research might explore whether the relationship between DSS and adiposity-related health outcomes varies by specific population subgroups, such as those defined by socioeconomic status or regional food availability.

The findings of this study may inform actionable strategies, particularly for policymakers seeking to translate evidence into meaningful, systemic changes. For example, policymakers may consider promoting sustainable food systems through funding for research and innovation in sustainable food production that prioritise nutrient-dense, minimally processed foods while making these diets economically and culturally accessible. Economic incentives, such as subsidies or tax breaks for sustainably produced nutrient-dense foods, may help reduce barriers to adopting dietary patterns similar to those associated with higher DSS, particularly for low- and middle-income populations, by lowering the financial barriers to adopting sustainable choices. Institutions such as schools, hospitals, and government facilities can play a vital role by prioritising sustainable food procurement, offering locally sourced, seasonal, and plant-based meals that exemplify healthy, sustainable eating. These actions could influence dietary habits more broadly, as public institutions act as sustainability models in practice. Educational campaigns aimed at increasing public awareness about the importance of sustainable eating patterns may further support the adoption of dietary behaviours aligned with higher DSS, potentially leading to long-term improvements in the ecological impacts of current food systems and reductions in the prevalence of diet-related non-communicable diseases, without forgetting the affordability and cultural acceptance of diets. Policymakers could also adopt robust monitoring and evaluation systems to track the health, environmental, economic and social impacts of sustainable dietary guidelines, allowing for data-driven policy refinement over time, thus ensuring responsiveness to evolving public health, environmental and socioeconomic challenges. Together, these strategies may foster a shift toward sustainable dietary patterns that, as suggested by the present results, are associated with more favourable adiposity profiles at the population level.

## 6. Conclusions

Higher DSS was associated with reduced odds of overweight, obesity, and unhealthy central adiposity. From a policy perspective, the results of this study have significant implications. Incorporating overall sustainability into national dietary guidelines can help address multiple public health objectives simultaneously, including obesity prevention, non-communicable disease management, and environmental conservation.

## Figures and Tables

**Table 1:** Sample characterisation and the Diet Sustainability Score, total and across health outcomes—IAN-AF 2015–2016.

	Total n (%)	BMI Classes n (%)		WHtR Classes n (%)		DSS Mean (CI 95%)
		Under/Normal Weight (n = 935)	Overweight/Obesity (n = 1335)	Healthy Central Adiposity (n = 791)	Unhealthy Central Adiposity (n = 1470)	
Sex, n (%)						
Female	1088 (47.6)	522 (55.8)	558 (41.8)	479 (60.6)	597 (40.6)	12.19 (12.02, 12.36)
Male	1199 (52.4)	413 (44.2)	777 (58.2)	312 (39.4)	873 (59.4)	11.83 (11.66, 12.00)
Age group, n (%)						
18–34 years old	536 (23.4)	374 (40.0)	162 (12.1)	389 (49.2)	145 (9.9)	10.82 (10.60, 11.05)
35–64 years old	1295 (56.6)	463 (49.5)	831 (62.2)	375 (47.4)	913 (62.1)	11.83 (11.68, 11.98)
65–84 years old	456 (19.9)	98 (10.5)	342 (25.6)	27 (3.4)	412 (28.0)	13.88 (13.63, 14.12)
Education level, n (%)						
≤6th year of schooling	698 (30.6)	154 (16.5)	531 (39.8)	69 (8.7)	612 (41.7)	12.61 (12.39, 12.84)
7th–12th year of schooling	978 (42.8)	444 (47.5)	531 (39.8)	398 (50.4)	575 (39.2)	11.42 (11.24, 11.60)
>12th year of schooling	608 (26.6)	336 (36.0)	271 (20.3)	323 (40.9)	281 (19.1)	12.22 (12.00, 12.44)
Physical activity level, n (%)						
Inactive	468 (21.1)	382 (42.1)	691 (53.4)	317 (41.6)	751 (52.6)	12.05 (11.88, 12.22)
Moderately active	665 (30.0)	303 (33.4)	357 (27.6)	256 (33.6)	403 (28.2)	12.32 (12.09, 12.54)
Active	1084 (48.9)	222 (24.5)	245 (18.9)	189 (24.8)	275 (19.2)	11.37 (11.10, 11.63)
DSS, mean (CI 95%)	12.00 (11.88, 12.12)	11.90 (11.72, 12.09)	12.03 (11.87, 12.19)	11.65 (11.45, 11.84)	12.16 (12.00, 12.31)	-

DSS, Diet Sustainability Score. BMI, Body Mass Index. WHtR, Waist-to-Height Ratio. CI, Confidence Interval. Under/Normal weight: the BMI is <25.0 kg/m^2^. Overweight: the BMI is 25.0–29.9 kg/m^2^. Obesity: the BMI is ≥30.0 kg/m^2^. Healthy central adiposity: the WHtR is <0.5. Unhealthy central adiposity: the WHtR is ≥0.5.

**Table 2:** Association between the Diet Sustainability Score and health outcomes.

	**BMI Classes**		
	**Under/Normal Weight**	**Overweight/Obesity**	
		**Crude Model** **OR (CI 95%)**	Adjusted Model * **OR (CI 95%)**
DSS	Ref	1.01 (0.98, 1.04)	**0.91 (0.88, 0.94)**
	**WHtR Classes**		
	**Healthy Central Adiposity**	**Unhealthy Central Adiposity**	
		**Crude Model** **OR (CI 95%)**	Adjusted Model * **OR (CI 95%)**
DSS	Ref	**1.06 (1.03, 1.09)**	**0.91 (0.87, 0.95)**

* Model adjusted for: sex, age, education and level of physical activity. Binary logistic regression models were fitted, and odds ratios are presented per one-unit increase in the DSS. Model diagnostics included assessment of multicollinearity and goodness-of-fit. DSS, Diet Sustainability Score. BMI, Body Mass Index. WHtR, Waist-to-Height Ratio. Ref, Reference group. OR, Odds Ratio. CI, Confidence Interval. Under/Normal weight: the BMI is <25.0 kg/m^2^. Overweight: the BMI is 25.0–29.9 kg/m^2^. Obesity: the BMI is ≥30.0 kg/m^2^. Healthy central adiposity: the WHtR is <0.5. Unhealthy central adiposity: the WHtR is ≥0.5.

## Data Availability

The data supporting this study’s findings are available from the corresponding author upon reasonable request.
